# Novel *HexA* splice site mutations in a patient with late atypical onset Tay-Sachs disease: importance of combined NGS and biochemical analysis

**DOI:** 10.3389/fneur.2024.1400989

**Published:** 2024-10-04

**Authors:** Alina Bilyalova, Elena Shagimardanova, Airat Bilyalov, Marina Zaripova, Leyla Shigapova, Guzel Gazizova, Pavel Mazin, Bukina Tatiana, Oleg Gusev

**Affiliations:** ^1^Institute of Fundamental Medicine and Biology, Kazan Federal University, Kazan, Russia; ^2^Loginov Moscow Clinical Scientific Center, Moscow, Russia; ^3^Life Improvement by Future Technologies Institute, Moscow, Russia; ^4^Skolkovo Institute of Science and Technology, Moscow, Russia; ^5^Research Centre for Medical Genetics, Moscow, Russia; ^6^Endocrinology Research Center, Moscow, Russia; ^7^Intractable Disease Research Center, Graduate School of Medicine, Juntendo University, Tokyo, Japan

**Keywords:** Tay-Sachs disease, *hexA* gene, next-generation sequencing, neurology, RNA

## Abstract

Tay-Sachs disease (TSD) is a rare genetic disorder with diverse clinical manifestations, often leading to underdiagnosis due to symptom similarities with other neurological conditions. In this study, we aimed to identify the genetic mutations underlying late-onset TSD in a 27-year-old patient with progressive neurological symptoms. Whole-exome sequencing revealed two *hexA* gene mutations associated with TSD: a previously known variant, c.805G > A (p.Gly269Ser), and a novel splice-site mutation, c.346 + 2dupT. Through clinical assessments, genetic analysis, and functional investigations—including RNA sequencing and enzymatic activity assays—we confirmed the pathogenicity of the novel mutation. Our findings highlight the efficacy of advanced genomic technologies in diagnosing intricate genetic disorders and emphasize the significance of functional validation to confirm the effects of mutations. Identifying compound heterozygous mutations in the *hexA* gene also provides insight into Mendelian inheritance patterns. This case highlights the diagnostic challenges posed by overlapping clinical phenotypes and emphasizes the need for increased genetic awareness among clinicians. Accurate diagnosis of TSD has significant implications for patients and their families, allowing for informed genetic counseling and guiding clinical management decisions. While current treatment options are limited, timely and accurate diagnosis holds promise for future research and therapeutic interventions. This study highlights the value of a multidisciplinary approach in exploring the molecular basis of complex genetic diseases and informing clinical decisions.

## Introduction

Tay-Sachs disease (TSD) is the most common genetic disorder among GM2 gangliosidoses (1). It is caused by pathogenic mutations in the *hexA* gene, leading to a deficiency of the enzyme *β*-hexosaminidase A (HexA), which is responsible for processing GM2 ganglioside into GM3 ganglioside in lysosomes (2, 3). Depending on the remaining HexA activity level, TSD can manifest in several clinical forms: the acute infantile variant, which leads to early death at the age 4; the late-onset (or subacute) form, which allows survival into later childhood or adolescence, and the chronic form (or adult-onset) TSD (4).

Adult-onset TSD shows significant variation in the age of disease onset, with cases appearing as late as age 42, and presents with a wide range of clinical symptoms. Diagnosing late-onset TSD is particularly challenging due to its symptom overlap with other neurological disorders, such as spinal muscular atrophy (SMA), spinocerebellar ataxia (SCA), motor neuron disease (MND), and other neurodegenerative conditions, as well as the rarity of the disease in many populations.

Several well-described variants are commonly found in TSD patients, including c.1277_1278insTATC, c.1421 + 1 G.C, and c.805 G.A (p.G269S), which are prevalent among Ashkenazi Jews and the Indian population (5, 6). In the majority of TSD cases in Ashkenazi Jews, the condition is caused by an insertion in the beta-hexosaminidase alpha-chain gene, resulting in the synthesis of a non-functional protein (7, 8). Other notable mutations include a large 7.6 kb deletion in the French Canadian population and the c.571–1 G.T mutation in the Japanese populations (9, 10).

To date, 164 pathogenic and 134 likely pathogenic variants have been reported in the *hexA* gene, but no clear mutational hotspots have been found (11). As a result, performing point mutation analysis in routine clinical practice remains challenging for TSD. In this study, we present the second reported case of late-onset TSD in the Russian Federation, along with the identification of a novel causative mutation (12). We employed a combined next-generation sequencing (NGS) approach to analyze both mutational load and transcript data, followed by biochemical confirmation of the causative effect of the identified mutation’s variant pathogenicity. We suggest that next-generation sequencing at both high-throughput DNA and RNA levels is an effective tool for elucidating the hereditary background of TSD, including in cases where the diagnosis is uncertain.

## Materials and methods

### Study subjects and ethical statement

A family consisting of two siblings and their parents participated in this study. All participants provided written informed consent before the collection of blood samples, documentation of pedigree structure, acquisition of clinical data, and explanation of laboratory findings.

### DNA extraction, exome enrichment, and variant call analysis

DNA was extracted from 200 μL of whole blood collected in a vacutainer with ethylenediaminetetraacetic acid (EDTA) using Qiagen Blood Mini Kit (Qiagen, Germany), following the manufacturer’s instructions. For next-generation sequencing, 100 ng of isolated DNA was used for library preparation using the NebNext DNA Ultra Directional I Kit (NEB, England). DNA fragmentation was performed through ultrasonication using Qsoniсa Q800R (Sonika, United States), resulting in a median fragment length of 200 bp. Fragment size distribution was assessed using an Agilent Bioanalyzer 2,100 (Agilent, Santa Clara, California, USA). Exome enrichment was conducted following the manufacturer’s protocol for the ‘NimbleGen SeqCap EZ Human Exome v2.0 (Roche NimbleGen Inc.). The enriched library was sequenced using the Illumina HiSeq2500 (Illumina, San Diego, CA) platform with paired-end reads (2 × 101 bp read length).

Raw reads were aligned to the human reference genome (hg19) using the BWA-MEM aligner (v0.7.15), with quality control performed using FastQC. Variant calling was conducted using GATK HaplotypeCaller (v3.6). The resulting Variant Call Format (VCF) files were annotated using SnpSift, SnpEff, ANNOVAR, and Alamut Batch. Pathogenic mutations were identified through the HGMD Professional 2018.1 and ClinVar databases. Variants were filtered and prioritized based on their pathogenicity scores (>0.95) obtained from Polyphen-2, MutationTaster, and CADD (>20). Furthermore, the variants were cross-referenced with the Human Gene Mutation Database (HGMD, http://data.mch.mcgill.ca/phexdb), with particular focus on genes associated with neurological disorders.

### Sanger sequencing

To validate the mutations identified through whole-exome sequencing in the patient, two primer sets (HEXA1: forward CCTTGGGCTTCTTTCTTT, reverse GAGTCTTGTGGGCATTTT; and HEXA2: forward AGTGAAGAGCCAGTGTGA, reverse AGGAGAAGAGGGGCACAA) were designed using Vector NTI software. These primers were designed to target the regions located upstream and downstream of each mutation and were used in a polymerase chain reaction (PCR) with Q5 NEB High Fidelity Master Mix (NEB, England). The PCR amplification protocol involved an initial denaturation at 98°C for 30 s, followed by 35 cycles of 10 s of denaturation at 98°C, 20 s of annealing (61°C for the first primer pair and 66°C for the second primer pair), and 20 s of extension at 72°C. A final extension was performed at 72°C for 2 min.

The PCR products were electrophoresed on a 1% agarose gel along with the appropriate negative controls and a DNA ladder. After purifying the amplicons using the QIAquick PCR Purification Kit (Qiagen, Germany), they were then sequenced via Sanger sequencing using the ABI Prism 3,500 (Applied Biosystems, CA, United States). The presence of the variants was confirmed using CLC Main Software.

### RNA-seq library preparation and sequencing

After collecting into a citrate-containing vacutainer, 200 μL of whole blood was separated into cellular and plasma phases by centrifuging for 10 min at 5000 g at 4°C. To the cell phase, 1 mL of TRIzol Reagent (Ambion Inc.) was added, and the samples were then frozen and stored at-80°C until further use. RNA was extracted using TRIzol Reagent following the manufacturer’s instructions. The quality of the total RNA was assessed using the Bioanalyzer 2,100 (Agilent), while the quantity and purity of RNA were measured using a NanoPhotometer (Implen).

Then, mRNA was isolated from 700 ng of total RNA, which had an RNA integrity number (RIN) of ≥7, using the NEBNext^®^ Poly(A) mRNA Magnetic Isolation Module (New England Biolabs). cDNA libraries were prepared using the NEBNext^®^ Ultra^™^ Directional RNA Library Prep Kit for Illumina (New England Biolabs), according to the manufacturer’s instructions. The size selection of the amplified libraries was performed using the BluePippin system (SAGE Science). The library quality was verified using the Bioanalyzer 2,100 (Agilent), and the yield was quantified via quantitative polymerase chain reaction (qPCR). The libraries were then sequenced on the HiSeq 2,500 platform (Illumina) with paired-end 125 bp reads.

### RNA-seq data analysis

The reads were mapped to the human genome (GRCh38) using HISAT2 (v2.1) with the parameters “-no-softclip-max-intronlen 1,000,000.” Information about splice sites and exon positions from Ensembl v93 was included in the genome index. The number of read pairs that unambiguously mapped to exon-exon junctions, specifically connecting either the first and second exons or the first and third exons, was quantified using Samtools. Coverage plots were then generated using a custom script in R, using the GenomicAlignments package.

### Enzymatic activity assay

Total *β*-hexosaminidase and β-hexosaminidase A activities in serum were measured as previously described (11), using the fluorogenic substrates 4-methylumbelliferyl-2-acetamido-2-deoxy-b-D-glucopiranoside (Sigma-Aldrich, St. Louis, MO, United States) and 4-methylumbelliferyl-N-acetyl-b-D-glucosaminide-6-sulfate (Glycosynth, Warrington, UK), respectively.

## Case presentation

### Clinical characterization of the patient

A 27-year-old patient presented with constant progressive limb weakness, slurred and stretched speech, impaired coordination, and difficulty walking. The patient showed no abnormalities in physical and psychological development until the age of 3. At the age of 3, the patient’s parents noticed slight weakness in the legs and impaired walking. By age 9, the patient’s speech had become slurred and stretched, an abnormal gait had developed, leg weakness had worsened, and handwriting had deteriorated. At the age of 23, the patient started using a wheelchair due to pronounced leg weakness. Then, 2 years later, the patient reported increased sensitivity, causing even light touches painful. Knee reflexes had disappeared. However, intellectual function remained intact, although adiadochokinesia and athetosis of the upper extremities were observed.

An MRI scan revealed significant atrophy (volume loss) in all cerebellar compartments of the brain, while the brainstem, supratentorial ventricles, sulci, and brain parenchyma appeared normal. At the age of 14 years. The patient received a genetic consultation for suspected hereditary ataxia, followed by genetic testing using a hereditary ataxia autosomal recessive multigene panel, which included the following genes: *ADCK3, APTX, FXN, SACS,* and *SETX*. However, the test did not reveal any pathogenic gene variants. Despite the onset of clinical symptoms at 9–10 years, an accurate diagnosis was not established.

### Identification of pathogenic mutation candidates

Since the disease had not been clearly diagnosed, a genetic cause was strongly suspected. We performed whole-exome sequencing on the patient’s DNA. Exome capture and sequencing using the Illumina Hiseq 2,500 platform generated 24,478,328 paired-end reads of 101 nucleotides. These reads were aligned to the human reference genome (hg 19), resulting in an average coverage depth of x 54.

In the patient’s case, two mutations were identified in the *hexA* gene. The mutation с.805G > A (p.Gly269Ser) had previously been characterized as a founder mutation in Ashkenazi Jews (7), which is associated with late-onset TSD in the homozygous state, or as compound heterozygosity with a null allele (13).

The second mutation c.346 + 2dupT is a newly identified mutation causing a double T insertion at position chr15:72648863 (HG19) in the second intron, potentially disrupting the RNA splicing process. This variant has not been previously reported in the Exome Aggregation Consortium database (14), the Human Gene Mutation Database (15), or the 1,000 Genomes Project (16).

To assess [u5] the effect of this splice site mutation, we conducted an *in silico* analysis to estimate the probability of splicing disruption. The PolyPhen-2 pipeline predicted a high pathogenicity prediction score, indicating that the mutation is highly likely to be pathogenic. Both variants were found to be heterozygous in the patient and were confirmed through Sanger sequencing.

**Figure 1:**
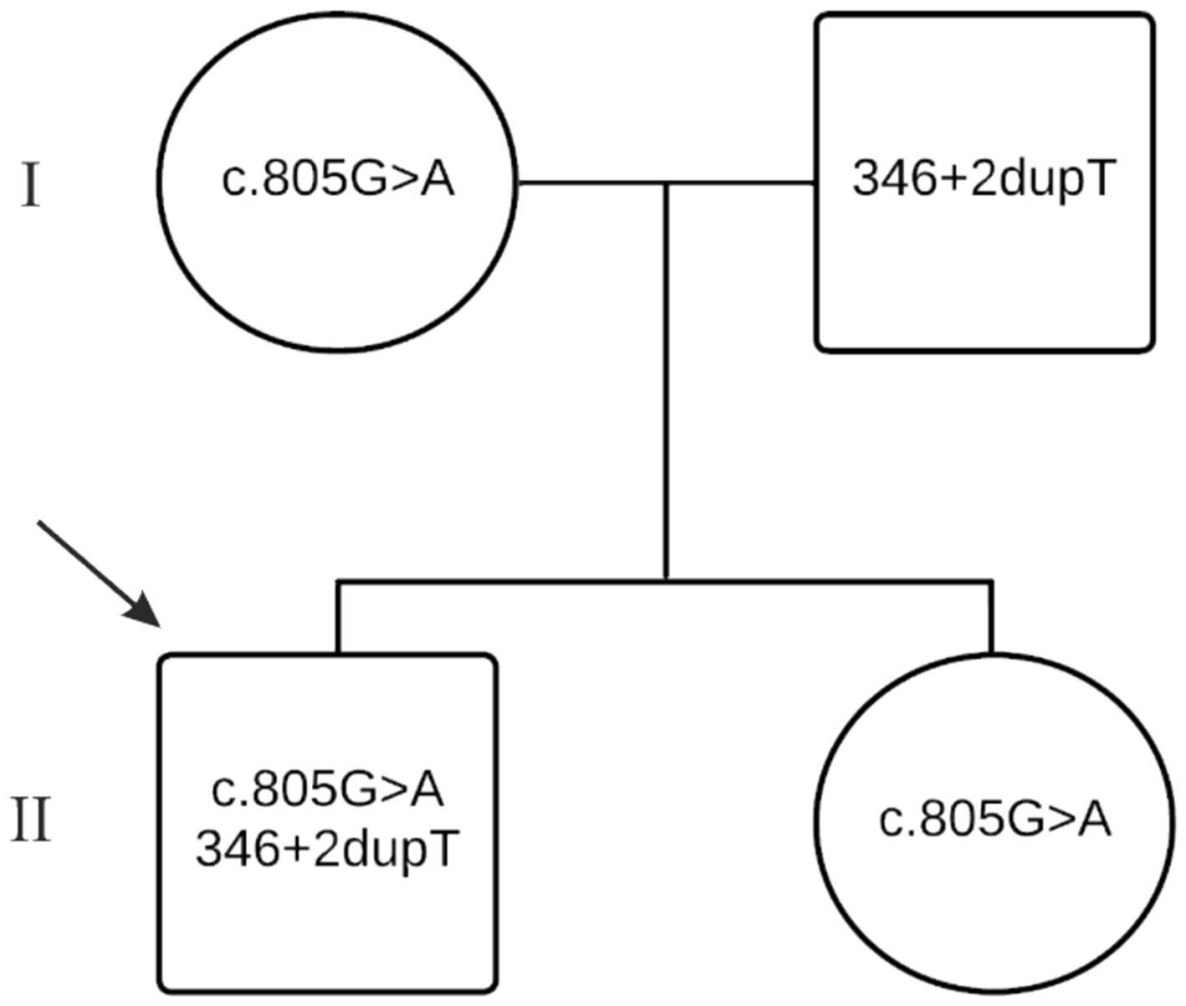
Family pedigree showing the respective genotypes of the parents and siblings.

Genetic testing of the *hexA* gene was also performed on DNA isolated from the patient’s parents’ and sister’s leucocytes using direct Sanger sequencing. The patient’s mother and sisterwere found to carry the с.805G > A variant, while the father was a carrier of the c.346 + 2dupT mutation. This analysis confirmed that the patient inherited one pathogenic allele from the mother and the other from the father, thereby confirming compound heterozygosity (Figure 1).

### Evaluation of mutation effects on splicing and enzymatic activities

To confirm the pathogenicity of the identified mutations, we analyzed the *hexA* gene’s products at both the RNA and protein levels in all family members. According to the FANTOM database (17), the *hexA* gene is highly expressed in the blood cells. Therefore, to assess the effect of the mutation on the splicing process, we performed whole transcriptomic sequencing of total RNA isolated from the patient’s, his parents’, and his sibling’s whole blood. RNA sequencing using the HiSeq 2,500 platform generated 26,729,711, 25,070,308, 31,328,028, and 25,845,035 paired-end reads for the patient, mother, father, and sister, respectively.

**Figure 2:**
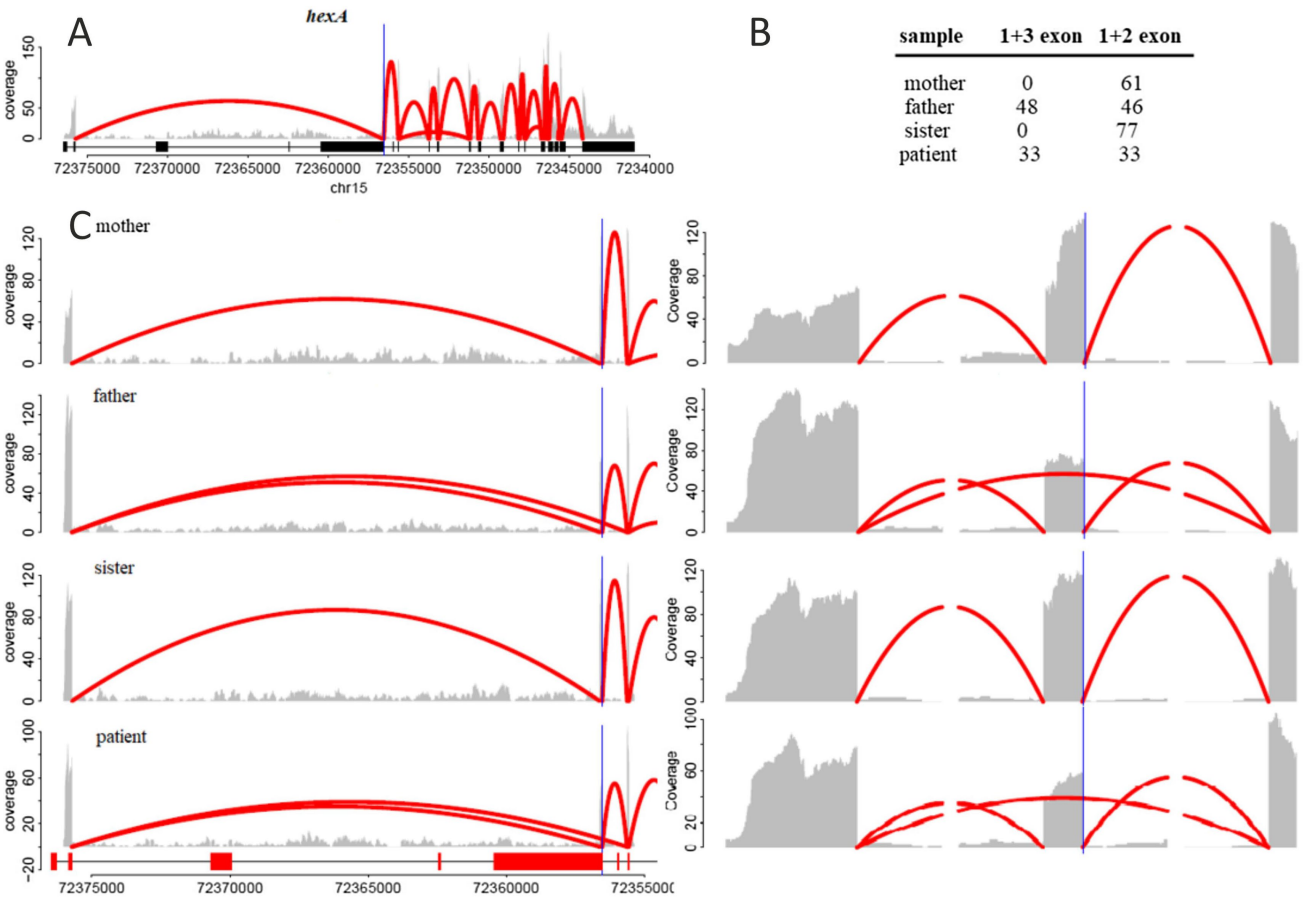
**(A)** RNA-seq reads coverage across the entire *hexA* gene. **(B)** Number of reads mapped to the junction between the first and third exons (indicating skipping of the second exon) and the number of reads mapped to the junction between the first and second exons (indicating the inclusion of the second exon). **(C)** Zoomed-in view of exons 1, 2, and 3. The gray area represents the number of reads mapped to the corresponding genomic position, while the red arcs indicate reads mapped to exon-exon junctions, with the number of reads represented by the maximal arc height.

After mapping the reads, we evaluated the number of wild-type and altered transcripts in all samples. RNA-Seq analysis showed that approximately half of the *HEXA* mRNA in the patient’s and father’s samples skipped exon 2, leading to a complete loss of this exon 2 (Figures 2B,C). This exon skipping likely results in impaired *HEXA* enzyme activity due to significant alterations in the resulting protein product. In contrast, the RNA-Seq analysis of the mother’s and sister’s samples showed that all *HEXA* mRNA was wild-type (Figure 2).

To confirm the sequencing results at the protein activity level and to verify the diagnosis, we performed a *HEXA* enzymatic activity assay (Table 1). The patient’s remaining hexosaminidase A activity was only 3.6% compared to the reference range of 30.9 to 72%. The enzyme activity levels in the mother, father, and sister were 36.9, 32 and 36.4%, respectively. This functional analysis confirmed the significant deficiency of *HEXA* enzymatic activity in the patient, providing strong evidence for the pathogenicity of the novel c.346 + 2dupT variant in the context of compound heterozygosity.

**Table 1:** Hexosaminidase A activity in blood serum.

	Patient	Sister	Mother	Father	Normal range
Hexosaminidase total activity (nmol/ml/h)	792	1,030,1	1,155,8	1,275,8	523–1,865
Hexosaminidase A activity (nmol/ml/h)	2,1	36,4	36,9	32	30,9–72

## Discussion

In this study, we aimed to identify the genetic cause of inherited ataxia in a patient who could not be accurately diagnosed based on clinical symptoms or various clinical tests. Using whole-exome sequencing, we identified two mutations in the *hexA* gene, one of which is novel. The alteration in the gene’s nucleotide sequence is known to impair the enzymatic function of hexosaminidase A, initiating a pathological process that leads to the accumulation of toxic GM2 gangliosides, thereby causing severe somatic symptoms. We did not find any other pathogenic or likely pathogenic variants in any other gene that could explain the patient’s symptoms.

One of the identified variants, с.805G > A, is a well-known mutation associated with the adult-onset form of TSD in Ashkenazi Jews. The second variant c.346 + 2dupT which likely disrupts a donor splice site, had not been previously reported, and its pathogenicity was unclear. To support the pathogenicity of this novel *HEXA* variant, we performed both an *in silico* analysis and a functional *in vitro* study. The *in silico* analysis suggested that this variant disrupts correct splicing at the second exon–intron junction.

Genetic testing of other family members showed that the patient’s mother and sister carry the с805G > A variant, while the father carries the c.346 + 2dupT variant, demonstrating Mendelian inheritance, which, in turn, May explain why none of the family members, except for the patient, exhibit the illness phenotype.

The RNA-seq analysis confirmed that the c.346 + 2dupT variant disrupts the splicing process, leading to transcripts lacking the second exon. RNA samples from the patient and his father demonstrated that approximately 50% of transcripts were spliced incorrectly, with the second exon skipped. It is worth noting that exon2 encodes a part of the beta-hexosaminidase domain, which is essential for *HEXA* activity (18).

Additionally, the RNA-seq data revealed a known pathogenic variant in exon 7, rs121907954 (chr15:72642859, NM_001318825.1:.838G > A), in all samples except for the father’s. In the patient, one allele carries the pathogenic c.805G > A variant, while the second allele lacks the second exon, severely compromising *HEX* A gene activity. In contrast, the father’s second allele remains intact, allowing for the production of wild-type transcripts and normal enzyme synthesis.

Finally, the enzymatic activity assay showed only traces of *HEXA* activity in the patient’s blood, whereas the enzyme activity levels in the blood samples of the mother, father, and sister were within the normal range. The significant reduction in protein activity in the patient provides strong evidence for the pathogenicity of compound mutation.

The case report described in this study clearly highlights the critical role of whole-exome sequencing in achieving an appropriate diagnosis. The patient’s symptoms did not allow clinicians to initially suspect TSD, resulting in a delay of more than 6 years before the correct diagnosis was made. A similar case of late-onset TSD in the Russian Federation took more than 13 years to diagnose (12). The rarity of the disease, especially its atypical forms, compounds the challenge, as it often leads to a lack of clinical expertise. For instance, even after identifying the pathogenic *HEXA* gene variant, an ophthalmological examination revealed no cherry-red spot characteristic of TSD.

Several aspects strongly suggest that the novel *hexA* gene variant is indeed pathogenic:

1. The presence of a compound mutation in the patient, with a clear inheritance pattern of two damaged alleles, one from each parent;
2. Functional studies at both the RNA and protein levels, which provided strong evidence of the variant’s pathogenicity; and
3. The absence of this variant in population databases.

Thus, whole-exome analysis revealed a novel mutation in the *hexA* gene and aided in clarifying the clinical diagnosis in a patient with hereditary ataxia of unknown etiology. Currently, no optimal therapy exists for TSD. Clinical trials for bone marrow transplantation and enzyme replacement therapy have been unsuccessful. However, establishing an accurate diagnosis offers significant benefits for both the patient and their family by eliminating the need for unnecessary diagnostic procedures and reducing financial costs. Moreover, a clear and precise diagnosis enables family members to seek genetic counseling for informed reproductive decisions.

This case, along with several other reports of late-onset TSD, suggests that the disease May be under-recognized as a cause of neurodegenerative or ataxia-related symptoms (19, 20). There is a necessity to increase clinicians’ awareness about the disease, which will lead to a timely and correct diagnosis and the development of genetic counseling among variant carriers ideally resulting in a reduction of new TSD cases in the population.

## Conclusion

This study provides a comprehensive analysis of a late-onset TSD case, demonstrating the value of next-generation sequencing in diagnosing complex genetic disorders. By identifying both a known mutation (c.805G > A) and a novel splice site mutation (c.346 + 2dupT) in the *hexA* gene, we establish a strong genetic basis for the patient’s clinical symptoms. Functional assessments, including RNA sequencing and enzymatic activity assays, provide compelling evidence for the pathogenicity of the novel mutation, shedding light on its impact on hexosaminidase A activity.

This study highlights the significance of combining genetic analysis with functional validation to confidently determine the causative mutations.

The findings highlight the diagnostic challenges of disorders with overlapping clinical presentations and emphasize the importance of genetic awareness among clinicians. Furthermore, the accurate diagnosis not only informs the patient’s clinical management but also enables informed genetic counseling for the family. While therapeutic options for TSD remain limited, early and precise diagnosis holds valuable implications for future research and potential interventions. This investigation emphasizes the importance of a multidisciplinary approach in unraveling the molecular basis of genetic diseases and guiding clinical decision-making.

## Data Availability

The datasets presented in this article are not readily available because of ethical and privacy restrictions. Requests to access the datasets should be directed to the corresponding authors.
